# Ranking of characteristic features in combined wrapper approaches to selection

**DOI:** 10.1007/s00521-014-1620-2

**Published:** 2014-06-11

**Authors:** Urszula Stańczyk

**Affiliations:** Institute of Informatics, Silesian University of Technology, Akademicka 16, 44-100 Gliwice, Poland

**Keywords:** Feature ranking, Feature selection and reduction, Wrapper, Filter, Sequential backward search, Stylometry

## Abstract

The performance of a classification system of any type can suffer from irrelevant or redundant data, contained in characteristic features that describe objects of the universe. To estimate relevance of attributes and select their subset for a constructed classifier typically either a filter, wrapper, or an embedded approach, is implemented. The paper presents a combined wrapper framework, where in a pre-processing step, a ranking of variables is established by a simple wrapper model employing sequential backward search procedure. Next, another predictor exploits this resulting ordering of features in their reduction. The proposed methodology is illustrated firstly for a binary classification task of authorship attribution from stylometric domain, and then for additional verification for a waveform dataset from UCI machine learning repository.

## Introduction

In supervised learning in order to recognise objects from each other, to be able to successfully classify them to decision classes, firstly, we need to characterise these objects by some descriptive features. Their nature and number determine possible types of a classification system to be constructed and its performance. When there are too many features, when there are repetitions, or too much of an overlap in information conveyed by them, the classifier can suffer from it [17]. Knowledge about *relevance* or *redundancy* of individual attributes or their groups can be useful not only at a classifier’s design stage, when it is typically exploited for their selection, but also for already working solutions, to optimise them, to reduce some of features, to enhance understanding of performed classification [29].

In selection and reduction of attributes, to establish their relevance or redundancy, there can be employed either a filter, wrapper, or an embedded approach [27]. Filters work separately and independently on classifiers and their parameters or performance. They can use expert domain knowledge, if available, or some other indicators, defined functions, or measures of importance or relevance. Wrappers adapt a set of features to specifics of the exploited classification system, basing on some feedback from its work, typically the predictive accuracy [66]. In embedded approaches, selection is an inherent mechanism of inductive learning algorithm, incorporated in it, such as pruning in artificial neural networks [30], activated relative reducts in rough sets [43, 68], or choosing a variable for a branching node in a decision tree construction.

The paper presents a two-step methodology, within which in the pre-processing stage, a simple wrapper is used to establish a ranking of characteristic features through greedy sequential backward elimination procedures [24]. The resulting ordering of variables is next imposed on another predictor to reduce its features. When both classifiers share the same general characteristics in the proposed framework, there is constructed a combined wrapper; when they differ significantly, the structure can be seen as treating a wrapper as a filter, thus resulting in a combined wrapper-filter solution. The performance of classifiers is observed in the perspective of gradually decreasing numbers of characteristic features involved in pattern recognition.

In the research described, two different types of inducers were employed, rule-based and connectionist, namely decision algorithms inferred with dominance-based rough set approach (DRSA) [21, 22] and artificial neural networks with Multilayer Perceptron (MLP) topology [19]. These classification systems were exploited separately and in combinations, within the same type or hybrid solutions [61, 62].

The procedures are firstly illustrated for a binary authorship attribution, which belongs to computational stylistics, or stylometric, area, a study of writing styles based on quantitative rather than qualitative textual descriptors, aiming at author characterisation, comparison, and recognition [4, 5]. Next, for additional verification and to provide a kind of benchmark study, the methodology is applied to waveform dataset from UCI machine learning repository [8].

The paper is organised as follows. Section 2 addresses the issue of relevance of characteristic features and their ranking. The problem of variable selection and reduction is presented briefly in Sect. 3, and the proposed research methodology in Sect. 4. Section 5 provides short descriptions of the learning systems exploited in research, stylometric domain of application with details of input datasets and used features, and waveform dataset. Obtained research results are illustrated and discussed in Sect. 6, whereas concluding remarks are given in Sect. 7.

## Relevance of characteristic features and their ranking

Algorithms dedicated to feature selection and reduction often refer to a concept of relevance, which can be defined in a variety of ways as we can have many reasons for formulating such definition [12].

Intuitively speaking, when a feature is irrelevant, it can be disregarded as useless for the induction process, which is a definition by contradiction. On the other hand, not all relevant attributes are in fact needed for classification to work, they can be relevant in varying degrees, and this relevance could depend on the presence or the absence of other features in the considered set, hence it should always be examined in some clearly stated context [40].

Probably the most natural notion of relevance from the perspective of feature selection problems is that of *incremental usefulness*, when the presence of some feature results in increased performance of a classification system comparing to its absence.

### **Definition 1**

(*Incremental usefulness*) [41] For a given data sample DS, a learning algorithm LA, and a set of features $A$, feature $x_i$ is incrementally useful to LA with respect to $A$ if the accuracy of the hypothesis produced by LA for the set of features $A\cup \{x_i\}$ is higher than the one achieved for $A$.

The definition is formulated for a case when adding a feature to some considered subset increases the performance. It can be extended to include also elimination of variables as follows.

### **Definition 2**

(*Usefulness*) For a given data sample DS, a learning algorithm LA, and a set of features $A$, feature $x_i$ is useful to LA with respect to $A$ if the accuracy of the hypothesis produced by LA for the set of features $A\cup \{x_i\}$ is higher than the one achieved for $A$. Feature $a_i\in A$ is useful to LA with respect to $A$ when the accuracy of $\hbox {LA}(A)$ is lower than that for $A\setminus\{a_i\}$.

Both definitions require the performance to increase or decrease after adding or, respectively, removing some feature. In reality, it may happen that, instead of seeking this change in performance, it can be easier to detect these variables that are irrelevant or redundant, and enable to keep the predictive accuracy at the same level, which leads to the concept of *weak usefulness*.

### **Definition 3**

(*Weak usefulness*) For a given data sample DS, a learning algorithm LA, and a set of features $A$, feature $x_i$ is weakly useful to LA with respect to $A$ if the accuracy of the hypothesis produced by LA for the set of features $A\cup \{x_i\}$ is not lower than the one achieved for $A$. Feature $a_i\in A$ is weakly useful to LA with respect to $A$ when the accuracy of $\hbox {LA}(A)$ is not higher than that for $A\setminus\{a_i\}$.

While establishing the usefulness of individual features or their groups can be the goal in itself (since it increases understanding of features), it can also be employed for a *ranking* of attributes, essentially in the same manner as retrieved documents are ranked accordingly to their relevance to some search query [6].

### **Definition 4**

(*Ranking*) [24] Given a data sample DS, and a set of features $A$, for each attribute $a_i\in A$ a scoring function $S$ assigns the score, which reflects how valuable the feature is with respect to the output variable.

By convention, the high score of the ranking function indicates that a feature is valuable, and after application of the scoring procedure, all variables are sorted in decreasing order of $S(i)$. When attribute ranking is used to construct some classification systems, more and more variables of decreasing relevance are included in nested subsets (with progressively increasing cardinalities) that are taken into consideration [38]. When ranking is exploited in the process of feature reduction, the most deeply nested subsets of attributes include those with the lowest scores as we want to reject these elements which are least relevant.

## Feature selection approaches

The most natural goal of feature selection algorithms is to find these variables that are relevant and at the same time detect those that are irrelevant or redundant. For plenty of applications, the concepts under study can be described by very high numbers of attributes, while they can also be defined by significantly fewer or simpler characteristic features, which helps in understanding data [26]. Dimensionality reduction enables to lower requirements with respect to storage and computational power, and smaller input variable sets can result in shortened processing time, or improved performance.

Before execution of any feature selection procedure, several decisions must be made that bear heavily on the final outcome. A starting point in the feature space needs to be selected, and this point determines possible directions for search algorithms. Furthermore, organisation of the search, feature subset evaluation strategies, and some stopping criteria must be chosen [15].

The procedure that generates a set of attributes can start with the empty set and then add a single element (or maybe a group of them) at a time in forward selection [50]. Or, it can begin with some initial set from which features are subsequently eliminated in backward reduction. It may also commence execution with a non-empty set that is in turns expanded and reduced.

Forward selection may seem as an obvious choice since it should involve lower computational costs of learning as the majority of candidate subsets of attributes have low cardinalities. We start with many small sets which gradually increase in size, but at the same time, the number of sets falls down. In case of rule classifiers, with just few conditional attributes the process of induction of decision rules does not take a lot of time, and storage requirements are certainly not prohibitive [47]. Yet within such limited context the interaction of some feature with others and its influence on classification could be more difficult to observe and conclusions drawn with respect to its relevance could be misleading. What is more, unless the case is trivial, training of a connectionist classification system with just few inputs is much more trying. Fewer network inputs mean fewer neurons which work as small and simple processing units. With their number being insufficient, the network can run into trouble and have noticeable difficulty with converging and then generalisation for unknown data [19].

In sequential backward reduction, the features and their relevance are observed in the presence of others and this wider context can be more advantageous; however, the initial dimensionality can be so high as to make the whole process unfeasible [1], as in this case the minority of sets are of lower cardinalities. Many attributes cause much higher number of decision rules to be inferred, and we start with correspondingly many such systems to be evaluated before the number of features decreases. On the other hand, it is far easier to have even more than necessary inputs to the artificial neural network as it learns quickly and the training rule is responsible for assigning the best weights to interconnections and by that degrees of relevance of inputs to the produced answer.

Search for some set of relevant attributes can be executed as a separate process, completely regardless of a classification system, in filtering approach, which then can be treated as some kind of pre-processing [25]. Features can be selected for example randomly, or referring to concepts of consistency, entropy, information gain [16]. Being general in nature, filters can be employed within any domain, for any learning system, yet most often at a cost of some lower predictive accuracy than available alternative solutions, which are not universal but adapted to specifics of a task under study.

If a selection strategy is conditioned by a learning process, the wrapper approach is used [33]. Wrappers exploit their own properties, especially their classification ratio, to estimate relevance of features, and by that suitability of the considered set for the particular task. Their close ties with classifiers result typically in improved performance but with the trade-off of some loss in generality, which can cause bias.

Embedded feature selection algorithms are intertwined with the learning processes, are their part, either explicit or implied [36]. When a wrapper has its own mechanism dedicated to variable selection and it is actively used, it becomes in fact an embedded solution. As examples from this category, there can be given decision trees where at each branching node a feature is chosen, artificial neural networks using pruning of input neurons [32], or rough set theory with activated relative reducts [46, 52].

A stopping point for a search procedure is to some extent determined by former choices with respect to the starting point, directions, and organisation of the search. Employing the concept of usefulness of features we can stop the search process when the system shows some significant and irreparable decrease in performance, if this is the primary goal of the selection process.

Alternatively, in forward selection, we can continue adding features, one after one, till the set of all available candidates is completely exhausted and we end with the full set of attributes, while in backward elimination, we can discard variables up to the time when we have only one left. These two extreme and opposite situations are mostly useful in observations of the overall inducer’s performance, when we want to try to find such smallest subset of variables for which the performance is the best (only when all subsets are tested we can confirm that some maximum is global and not local), or when detected characteristics in the feature set result in obtaining a ranking of variables, which can be employed for other inducers.

Feature evaluation, estimation of their individual or group relevance, ranking, selection and reduction procedures significantly gain in importance in cases when expert domain knowledge is missing or insufficient to establish relevance, and this task is transferred to data mining area [27]. Even when this expert knowledge is available, search for important features governed by principles of techniques and algorithms used to detect patterns in data can result in better understanding, knowledge discovery, uncovering new information and relationships [10, 18].

## Proposed research framework

The paper proposes a methodology that is a combination of feature selection approaches, while exploiting two types of learning systems (rule-based and connectionist), with the objectives of: (1) observing feature relevance and their usefulness through the process of their sequential backward elimination that leads to feature ranking, and next (2) using the obtained ranking in construction of other predictors.

The procedure consists of two subsequent phases:

1. Pre-processing ranking stage—for the initial arbitrarily selected set of characteristic features, there is executed scoring in backward reduction, basing on performance of an inducer. At each step, a single attribute is discarded, elimination of which resulted in the best classification accuracy among all candidate systems at this step. The stage ends when the set of variables is exhausted and the ordering in which they were eliminated gives base to establishing a ranking of all considered features.
2. Combined wrapper stage—following the ordering of attributes from the pre-processing stage that defines their ranking, nested subsets of features are taken out from the initial set, and for these remaining new predictors constructed. The processing stops when no variable is left to reduce.

Since by definition and execution, a ranking is a separate process from the learning algorithms induced in the second stage, following the general classification of approaches [33], we can treat it as filtering of features, which leads to wrapper-filter solutions. However, when classifiers from both steps share characteristics, it is rather a combination of two wrappers.

Within the pre-processing stage at $i$th step, $(N-i)$ new systems are built, $N$ being the initial number of variables. It means that overall the number of induced classifiers equals:

1 $$\begin{aligned} \sum \limits _{i=0}^{N-1}(N-i)&= N+(N-1)+(N-2)+\cdots +2+1=\frac{(N+1)N}{2}. \end{aligned}$$

Depending on $N$ and the complexity of induction process, this number can become prohibitive and the procedures too time consuming. The execution can be sped up by observing that although the reduction stages need to be performed in sequence as we need results from one to attempt the next; within a stage, all candidate systems are independent on each other, which means that they can be induced and tested in parallel and only their results compared to make a final choice of an attribute to be eliminated.

In the second phase $N$ inducers are built, the first with the complete set of $N$ attributes, next with their gradually decreasing numbers till only a single variable remains in the input set.

## Experimental evaluation

In the research described in this paper, two distinctively different approaches to data mining were used, namely DRSA which infers rules that form decision algorithms, and a connectionist solution of artificial neural networks (ANNs) in MLP topology [70].

The usefulness of the proposed methodology was evaluated by application in the field of stylometry, a branch of science that involves analysis of writing styles and claims that they can be uniquely and unambiguously expressed by quantitative measures [49]. Author attribution is considered as the most important of stylometric tasks [69]. It combines author characterisation with comparison [14] and can be regarded as classification, binary or multi-class, depending on the number of compared authors [2].

For additional verification, the same procedures were next employed to waveform dataset from the popular UCI machine learning repository [8], to provide a benchmark study for comparisons.

### DRSA processing

DRSA was invented to support multi-criteria decision-making [57]. It is a modification of the original classical rough set approach (CRSA) that was defined by Pawlak [45].

DRSA observes monotonicity in values of both conditional and decision attributes, and instead of just discerning (or not) classified objects as CRSA does, it assumes that all values are more or less *preferred* and applies weak preference and dominance relations. Preferences in data sets are defined either with the help of expert domain knowledge, assigned arbitrarily, or adjusted through some additional algorithm [67]. Dominance allows not only for nominal, but also for ordinal classification.

DRSA procedures induce decision rules through the process of reduction of excessive and redundant information in data sets with the help of rough approximations [37]. The sets to be approximated are dominance cones, corresponding to upward and downward unions of decision classes, and a rule classifies to either *at most* or *at least* some decision class.

The inferred rules consist of two parts: the premise, containing single or multiple conditions on individual attributes, which specify values either lower or equal, or higher or equal than the thresholds induced from all learning samples contained in the decision table; and decision parts:

2 $$\begin{aligned} \hbox{IF} \quad \hbox {cond}_1\; \& \; \hbox {cond}_2 \; \& \ldots \& \; \hbox {cond}_i \quad \hbox{THEN} \quad \hbox {at}\; \hbox {most} \;\hbox {decision}_s \end{aligned}$$

3 $$\begin{aligned} \hbox{IF} \quad \hbox {cond}_1\; \& \; \hbox {cond}_2 \; \& \ldots \& \; \hbox {cond}_i \quad \hbox{THEN} \quad \hbox {at}\; \hbox {least} \;\hbox {decision}_s \end{aligned}$$

Many algorithms for induction of decision rules exist [9, 53], probably the fastest of which (but not the simplest) is generating only so many rules as to provide a minimal cover of the learning samples [42]. The opposite approach is to construct all rules on examples and then choose only some subset of them by imposing some hard constraints [64], for example a minimal support required that indicates for how many learning samples a rule is true, or a maximal rule length giving the number of conditions included in the premise [65]. Or, some group of rules is induced, neither minimal nor complete, then the process of their pruning or adjusting is executed [54, 55]. All these approaches offer higher chances of good recognition ratio, yet computational costs involved could be significant and should be weighted against possible gains [23].

### ANN classifier

Multilayer Perceptron (MLP) is a unidirectional, feed-forward artificial neural network, with neurons grouped into some number of layers. It accumulates knowledge from the training samples using some learning rule, which determines how to adjust weights of interconnections in order to get the value on the network output as close as possible to the one that is expected. Popularly, there is used some version of backpropagation algorithm which minimises the error on the output, calculated as a difference between the desired and actually received value, for all outputs and all training samples [19].

In the research, California Scientific Brainmaker software for simulation of neural networks was used. To lower the influence of initiation of weights on the learning phase, multi-starting approach was employed and each network trained several times with randomising weights before each training, with noting the worse, average, and the best performance. In each case, the structure (established through tests) contained two hidden layers, with the total number of neurons in them equal to the cardinality of the currently considered set of characteristic features. The network outputs corresponded to recognised decision classes.

### Stylometric features

Categorisation of a text with respect to the subject content requires searching for some matching key words or phrases [11]. Authorship attribution means categorisation by authors, which is more challenging because we need to recognise and discern specific styles of writing and a writing style is not conveyed in *what* we write about, but in *how* we do it [35].

Features describing styles need to refer to such elements that are not easily imitated or common to many authors, reflect individual linguistic preferences, whether conscious or subconscious, observable in many samples [3, 34]. Popularly, there are exploited either lexical or syntactic descriptors, the first providing some statistical characteristics such as average word length, average sentence length, frequencies of usage for characters, words or phrases, distributions of all these averages and frequencies [44], while syntactic markers refer to punctuation marks and the way in which they organise the structure of the text into units of sentences, paragraphs [7]. These descriptors need to be calculated over many examples, using sufficiently wide corpus, otherwise they would be unreliable [39].

By the very definition, all writing styles are unique for their authors; hence, even though stylometry suggests some types of candidate characteristic feature sets, there is no one and only universal rule how to construct them, which would be applicable in all possible cases, for all writers, and regardless of techniques employed [13, 48]. Instead, many sets of descriptors are studied and adapted to specifics of the particular task under consideration. In the same way, several processing techniques are employed, typically either statistic-oriented computations [31, 51], or methodologies belonging with artificial intelligence domain [28, 61].

In the research presented in this paper as texts to study, there were taken literary works of Jane Austin and Edith Wharton, available in several electronic formats for download and online reading due to Project Gutenberg (http://www.gutenberg.org). The novels were divided into smaller parts of comparable size. For both learning and testing sets, one-half could be attributed to one author and the second to the other, giving perfectly balanced data sets.

For all these text samples, there were calculated frequencies of usage of 25 linguistic elements:

- 17 function words—but, and, in, with, what, for, from, by, not, that, to, of, this, if, at, on, as,
- 8 punctuation marks—a full stop, a comma, a colon, a semicolon, a question mark, an exclamation mark, a bracket, a hyphen,

employed in the earlier research on authorship attribution [60, 63]. The attributes obtained that way have real values, which needs to be taken into account while choosing some data mining technique, but of course we can also employ some discretisation strategy [18, 58].

### Waveform dataset

UCI machine learning repository is a popular source of datasets used as kind of benchmark studies for comparison. To make the classification task comparable to the one of previously described binary authorship attribution, from all available datasets, the one named Waveform Database Generator (Version 1) was selected. As it is many times larger than the stylometric dataset, only a part was involved in executed tests.

The number of attributes considered is 21, and there are three decision classes corresponding to three types of recognised waves. Once the complete set of 5,000 samples was put in increasing order with respect to these classes, for type 0 and type 1, first one hundred of samples were taken to be included in the learning dataset and the next fifty for the training set. In this way, also for these datasets, the classification becomes binary and samples for decision classes balanced.

## Research results

The experiments conducted within the described research were executed in two stages. In the first stage, the sequential backward elimination (SBE) algorithm, applied in the wrapper model, was used to establish ranking of characteristic features, revealing their relevance. The wrapper was constructed for two types of classifiers, minimal cover decision algorithms (MCDA) inferred in DRSA and artificial neural networks.

The two obtained rankings were next employed in the second stage, where reduction of attributes was performed, again for rule and connectionist inducers, while their performance was observed. The elimination of variables for DRSA classifier at this stage was executed in two ways: by discarding attributes and inducing new rules and algorithms, and by rejecting rules from the previously generated full decision algorithm (FDA), with all rules on examples, inferred for all features considered.

The procedures were applied to two pairs of datasets. The primary classification task was binary authorship attribution with stylometric features. For comparison sake, the tests were also executed for waveform dataset with similar characteristics (the same number of classes, comparable numbers of samples and attributes). The results for this second dataset are given at the end of this section.

### Establishing ranking of features by SBE

Since DRSA classifier was to be used as a wrapper with sequential backward reduction of features, it meant starting with the complete set of attributes and elimination of one element at a time. Hence, induction of all rules on examples in each case would be impractical as for 25 features in the FDA algorithm, there were 62,383 constituent decision rules. Instead, minimal cover decision algorithms MCDA were inferred and their performance used to select an attribute, reduction of which gave the best results when compared to others at the same level. The details for all steps are listed in Table 1, where the right-most column (i) shows the established DRSA Ranking of characteristic features.

**Table 1:** Backward elimination of attributes basing on the performance of DRSA classifiers

								DRSA Ranking
(a)	(b)	(c)	(d)	(e)	(f)	(g)	(h)	(i)
0	25	but and not in with on at of as this that by for to if what from . , ; : ! ? ( -	30		6	6	76.67	and
1	24	but not in with on at of as this that by for to if what from . , ; : ! ? ( -	30		2	17	77.78	!
2	23	but not in with on at of as this that by for to if what from . , ; : ? ( -	29		3	14	81.11	,
3	22	but not in with on at of as this that by for to if what from . ; : ? ( -	31		3	17	82.22	?
4	21	but not in with on at of as this that by for to if what from . ; : ( -	30		3	15	83.33	what
5	20	but not in with on at of as this that by for to if from . ; : ( -	30		3	13	85.56	:
6	19	but not in with on at of as this that by for to if from . ; ( -	30		3	13	85.56	.
7	18	but not in with on at of as this that by for to if from ; ( -	29		4	11	85.56	of
8	17	but not in with on at as this that by for to if from ; ( -	28		3	15	85.56	that
9	16	but not in with on at as this by for to if from ; ( -	26		3	16	85.56	(
10	15	but not in with on at as this by for to if from ; -	26		3	16	85.56	this
11	14	but not in with on at as by for to if from ; -	27		3	16	85.56	but
12	13	not in with on at as by for to if from ; -	24		2	17	86.67	if
13	12	not in with on at as by for to from ; -	23		2	16	86.67	at
14	11	not in with on as by for to from ; -	23		2	17	86.67	to
15	10	not in with on as by for from ; -	23		2	17	86.67	-
16	9	not in with on as by for from ;	23		2	18	86.67	with
17	8	not in on as by for from ;	22		2	20	86.67	on
18	7	not in as by for from ;	25		2	22	88.89	from
19	6	not in as by for ;	22		4	16	87.78	;
20	5	not in as by for	21		2	18	91.11	for
21	4	not in as by	17	15	7	11	91.11	in
22	3	not as by	18	10	10	10	91.11	as
23	2	not by	26	8	10	8	84.44	by
24	1	not	3	2	55	2	61.11	not

Columns present parameters: (a) elimination stage, (b) number of characteristic features left, (c) set of currently considered variables, (d) number of rules in DRSA minimal cover decision algorithm without any constraints, (e) number of exact rules when they are fewer than the total number, (f) minimal support required of DRSA rules resulting in maximal classification accuracy, (g) number of exact DRSA rules meeting constraints on support, (h) maximal predictive accuracy of the classifier (%), and (i) attribute selected to be eliminated

The top row of the table corresponds to the 0th reduction stage, that is the rule classifier induced for all 25 conditional attributes studied, listed in column (c). The minimal cover decision algorithm generated consisted of 30 constituent rules, which was limited to just 6 while demanding their minimal support to be equal at least 6. The maximal classification accuracy gained by the imposed constraint was 76.67 % of correctly recognised testing samples. Classification accuracy specified in the table (and for all other cases of data mining with DRSA presented in this paper) refers only to cases when all matching rules classified correctly. The ambiguous cases of contradicting decisions or no matching rules were always treated as incorrect (which is rather strict but limits additional processing needed otherwise).

Next, 25 new MCDA classifiers were constructed, each with 24 input features, with one attribute eliminated, and their performance tested and compared. Out of these systems, the one with the reduced feature corresponding to the frequency of usage for “and” gave the best result, so this attribute is selected as the least relevant of all candidates and the first to be eliminated, as given in column (i) of the table.

The set of 24 remaining variables gives base for the next reduction stage with index equal 1, shown in Table 1 in the second row. Again the best MCDA decision algorithm consisted of 30 rules, but with support equal or higher than 2, there were 17 rules with maximal classification reaching 77.78 %.

It can be observed in column (h) of the table that classification accuracy gradually increases from 76.67 % up to the maximum of 91.11 % correctly recognised samples when there are only 5, 4, or 3 features left in the input set, then to decrease to 84.44 % for two conditional attributes, and 61.11 % for a single attribute.

**Fig. 1:**
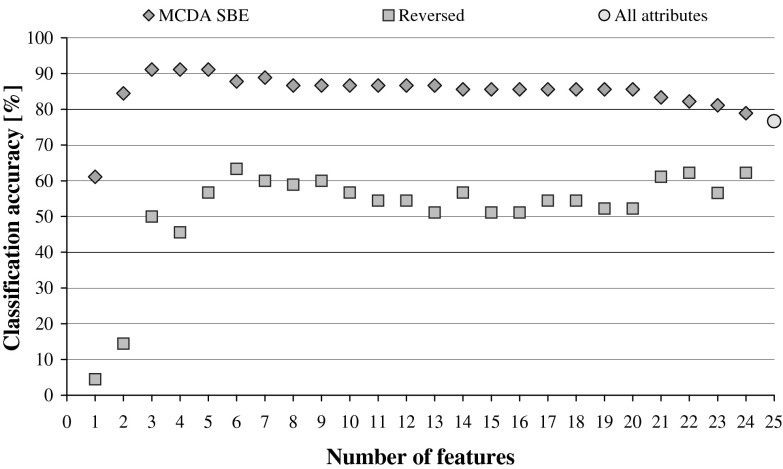
DRSA classification accuracy in relation to the number of features within sequential backward elimination with MCDA, compared with reduction of attributes using reversed ranking

The process of attribute elimination can be interpreted in this way that the system discards these elements that are irrelevant or redundant and keeps these that are essential for classification, as a result the classification accuracy either increases or is at least at the same level, but for fewer features. The order in which the attributes are eliminated reflects their importance. When this order is reversed, the performance of DRSA classifiers decreases immediately and irrecoverably, which is illustrated in Fig. 1.

The same sequential backward reduction procedure was next applied to ANN classifiers (Table 2), starting with constructing a network for all 25 features. For this set, the average classification accuracy was just above 91 %. This value is obviously higher than for the base DRSA classifier, for which it was only 76.67 %. However, it should be noted that the ambiguous classification of the rule-based system, of contradicting decisions or no rules matching, was treated as incorrect in all considered cases and that influenced this lower predictive accuracy. What is more, generation of minimal cover decision algorithms does not guarantee induction of the best rules, with the highest potential for correct classification, and it is quite common that decision algorithms constructed with other approaches test significantly better, yet at the cost of more complex procedures, more computational costs involved, and more processing time needed [56].

**Table 2:** Backward elimination of attributes basing on the performance of ANN classifiers

				ANN Ranking
(a)	(b)	(c)	(d)	(e)
0	25	but and not in with on at of as this that by for to if what from . , ; : ! ? ( -	91.11	,
1	24	but and not in with on at of as this that by for to if what from . ; : ! ? ( -	93.89	(
2	23	but and not in with on at of as this that by for to if what from . ; : ! ? -	94.44	-
3	22	but and not in with on at of as this that by for to if what from . ; : ! ?	95.56	at
4	21	but and not in with on of as this that by for to if what from . ; : ! ?	96.67	with
5	20	but and not in on of as this that by for to if what from . ; : ! ?	97.78	what
6	19	but and not in on of as this that by for to if from . ; : ! ?	97.78	from
7	18	but and not in on of as this that by for to if . ; : ! ?	97.78	to
8	17	but and not in on of as this that by for if . ; : ! ?	97.78	for
9	16	but and not in on of as this that by if . ; : ! ?	97.78	of
10	15	but and not in on as this that by if . ; : ! ?	97.78	.
11	14	but and not in on as this that by if ; : ! ?	98.89	in
12	13	but and not on as this that by if ; : ! ?	98.33	!
13	12	but and not on as this that by if ; : ?	98.89	this
14	11	but and not on as that by if ; : ?	98.89	but
15	10	and not on as that by if ; : ?	98.89	that
16	9	and not on as by if ; : ?	98.89	if
17	8	and not on as by ; : ?	97.78	?
18	7	and not on as by ; :	97.78	and
19	6	not on as by ; :	95.56	by
20	5	not on as ; :	94.44	:
21	4	not on as ;	95.56	as
22	3	not on ;	90.00	on
23	2	not ;	82.22	;
24	1	not	62.22	not

Columns present parameters: (a) elimination stage, (b) number of characteristic features left, (c) set of currently considered variables, (d) average predictive accuracy of the classifier (%), (e) attribute selected to be eliminated

The positive change of the classification ratio, or the same performance for fewer inputs is not the only indicator of attribute relevance or redundancy. When some feature is reduced, also the internal structure of the classifier is accordingly modified. For DRSA processing, it means fewer constituent rules in a decision algorithm, while for an artificial neural network, its layers get smaller by removal of neurons.

**Fig. 2:**
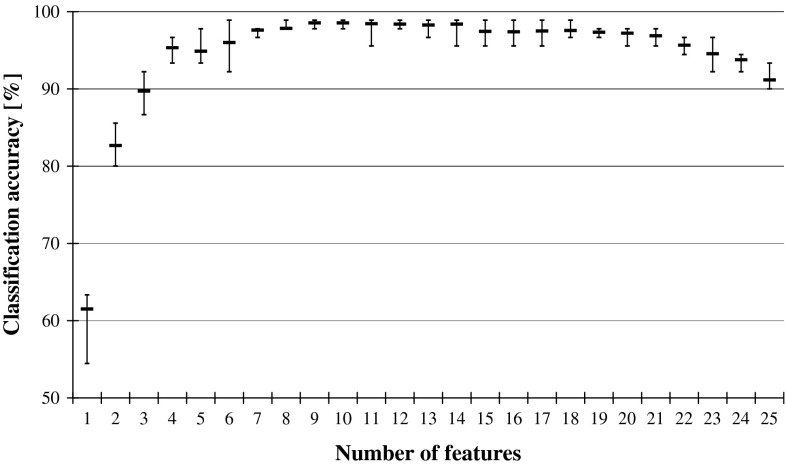
ANN classification accuracy observed in sequential backward elimination process, in relation to the number of considered features, and for each average, there is indicated maximal and minimal performance

**Fig. 3:**
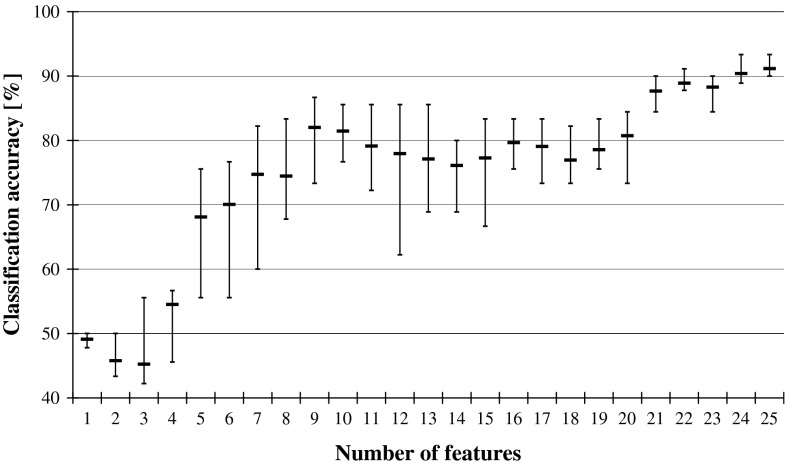
ANN classification accuracy in relation to the number of features, observed in backward reduction of inputs while following the reversed ANN Ranking. For each average, there is indicated maximal and minimal performance

If such smaller network classifies not worse than before reduction, it means that the relevance of the recently discarded input is negligible and it can be treated as redundant. The performance is illustrated in Fig. 2, while Fig. 3 shows what happens to the classification accuracy of the system when the input features are reduced while following the reversed ANN Ranking. The two graphs from Figs. 2 and 3 show the same trends that are visible in the previously plotted performance of DRSA classifiers in Fig. 1.

When we compare DRSA and ANN Rankings against each other, and analyse the scores assigned to all attributes, we can see that even though both types of classifiers operate on the same data sets, the resulting orderings of reduced features are different, only the last remaining feature is the same in both rankings: the frequency of usage for “not”. This is a direct result of the inherent characteristics of the inducers that are transferred to the rankings calculated with their help.

As wrappers are often accused of such bias, the obtained rankings need to be observed in the process of reduction of characteristic features for other classification systems, by combining wrappers of the same and different type, to evaluate their usefulness through tests, which is illustrated in the next section.

### Employing ranking of features in their reduction

Following the general categorisation of feature selection approaches [33], ranking belongs with filters. In the research presented, two rankings were obtained using DRSA- and ANN-based wrappers, given in the right-most columns of Tables 1 and 2. These orderings were next used to filter out the conditional attributes from the original set of 25, in backward elimination of input variables for new classifiers.

The details of application of ANN Ranking to backward reduction of attributes in DRSA processing, which results in a hybrid solution, are shown in Table 3. Firstly, subsets of features with increasing cardinalities were rejected, and then for the remaining subsets, new decision algorithms were induced, with providing just a minimal cover MCDA, and also with inferring all rules on examples FDA.

**Table 3:** Backward elimination of conditional attributes using ANN Ranking with induction of new decision algorithms

		Induction of DA after attribute elimination									
		Minimal cover DA					All rules on examples DA				
(a)	(b)	(c)	(d)	(e)	(f)	(g)	(c)	(d)	(e)	(f)	(g)
1	24	44		2	22	71.11	55,418		61–62	21	86.67
2	23	44		2	24	71.11	44,836		61–62	21	86.67
3	22	44		2	24	71.11	37,881		61–62	21	86.67
4	21	44		2	24	71.11	29,401		61–62	21	86.67
5	20	40		2	29	67.78	23,146		61–62	21	86.67
6	19	42		2–3	20	67.78	18,325		61–62	21	86.67
7	18	40		3	20	71.11	13,693		61–62	20	86.67
8	17	39		2–3	19	71.11	10,495		61–62	20	86.67
9	16	32		6–8	5	77.78	7,214		61–62	16	85.56
10	15	30		6–33	4	75.56	5,066		61–62	16	85.56
11	14	31		2	25	77.78	3,535		61–62	16	85.56
12	13	35		2, 4–11	6	67.78	2,534		61–62	16	85.56
13	12	28				75.56	1,822		61–62	15	85.56
14	11	31		1–7	8	71.11	1,197		55–62	11	84.44
15	10	29		4–7	11	78.89	636		55–62	11	84.44
16	9	21		4–11	11	78.89	433		55–62	11	84.44
17	8	18		1–10	7	78.89	311		55–62	11	84.44
18	7	20		1–10	7	76.67	199		55–62	11	84.44
19	6	20		1–3	18	84.44	109		55–62	11	84.44
20	5	13		25	6	83.33	40		55–62	10	84.44
21	4	26	10	6–7	7	81.11	72	22	12–20	14	78.89
22	3	25	4	5–34	3	65.56	18	11	3–31	8	65.56
23	2	22	3	5–54	7	61.11	7	5	1–55	3	61.11
24	1	3	2	1–54	2	61.11	3	2	1–55	2	61.11

		Minimal cover DA					All rules on examples DA				
(a)	(b)	(c)	(d)	(e)	(f)	(g)	(c)	(d)	(e)	(f)	(g)
0	25	30		6	6	76.67	62,383		65–66	17	86.67

Columns present parameters: (a) elimination stage, (b) number of characteristic features left, (c) number of all rules in a decision algorithm, (d) number of exact rules in a decision algorithm when they are fewer than the total number of rules, (e) value or range of values for minimal support required of rules resulting in maximal classification accuracy, (f) minimal number of rules meeting constraints, and (g) maximal classification accuracy (%)

**Fig. 4:**
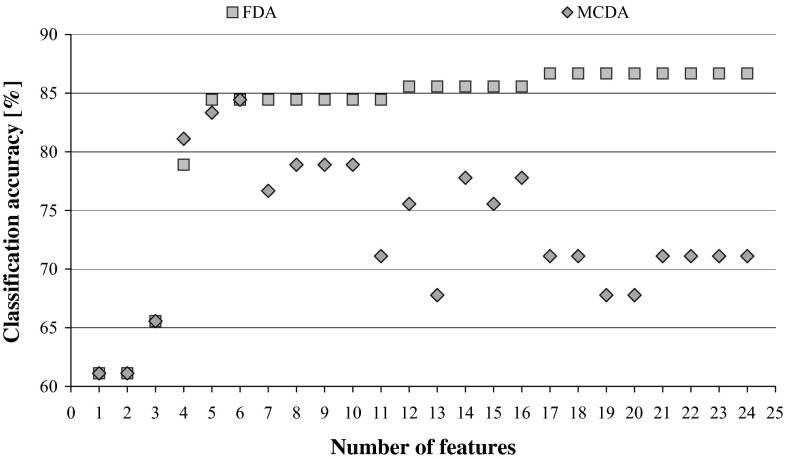
Classification accuracy for MCDA and FDA decision algorithms induced after backward attribute elimination based on ANN Ranking, in relation to the number of features

Since the classification accuracy is usually treated as the most important factor indicating the quality of the obtained solution, we can focus our attention on two (g) columns in Table 3, or a graph in Fig. 4. For both MCDA and FDA classifiers, there are several cases of improved or the same performance when features are reduced, yet the gain, considered in terms of either a number of rejected features, or an increase in predictive accuracy, or a lower number of decision rules remaining in the algorithm, is not so high as it was observed previously for simple ANN or MCDA wrappers.

Instead of reducing conditional attributes and then inferring new decision algorithms, which can be very time consuming, we can also eliminate these attributes by discarding rules with conditions on them, limiting all rules on examples decision algorithm induced previously for all features [59, 63]. Such approach can be considered as execution of ranking for decision rules.

Firstly, to each rule in the induced algorithm, a score is assigned, basing on individual scores for all attributes included in the premise part of the rule. From all these elementary scores, corresponding to constituent conditions, the highest one is chosen, indicating the attribute that is perceived as the least important; thus, the first to be eliminated, and this score is given to the decision rule. Then all rules are ordered by their scores, and in each step of reduction, all rules with a certain score are rejected, which results in reduced decision algorithms.

**Fig. 5:**
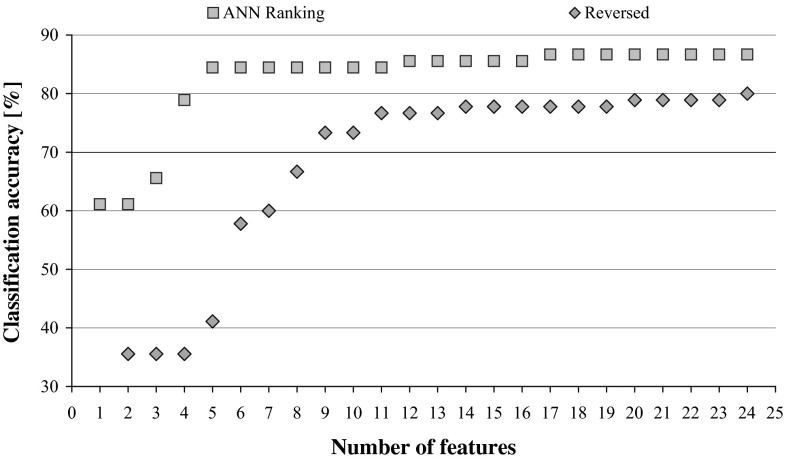
Reduction of FDA algorithm while following ANN Ranking and its reverse. The predictive accuracy is plotted in relation to the number of features

The details of this decision rule ranking procedure are given in Table 4. For comparison, there are also listed results of FDA algorithm reduction while following the reversed ANN Ranking, both plotted also in Fig. 5.

**Table 4:** Reduction of all rules on examples algorithm (FDA) using ANN feature ranking and its reverse

		ANN Ranking					Reversed				
(a)	(b)	(c)	(d)	(e)	(f)	(g)	(c)	(d)	(e)	(f)	(g)
1	24	,	55,418	61–62	21	86.67	not	61,382	48	49	80.00
2	23	(	44,836	61–62	21	86.67	;	56,666	38	35	78.89
3	22	-	37,881	61–62	21	86.67	on	51,063	38	35	78.89
4	21	at	29,401	61–62	21	86.67	as	40,112	38	28	78.89
5	20	with	23,146	61–62	21	86.67	:	36,947	38	28	78.89
6	19	what	18,325	61–62	21	86.67	by	28,085	38	20	77.78
7	18	from	13,693	61–62	20	86.67	and	20,140	38	20	77.78
8	17	to	10,495	61–62	20	86.67	?	17,000	38	20	77.78
9	16	for	7,214	61–62	16	85.56	if	13,272	38	19	77.78
10	15	of	5,066	61–62	16	85.56	that	10,711	38	18	77.78
11	14	.	3,564	61–62	16	85.56	but	7,666	38	13	77.78
12	13	in	2,580	61–62	16	85.56	this	5,265	21	71	76.67
13	12	!	1,880	61–62	15	85.56	!	3,678	21	58	76.67
14	11	this	1,239	55–62	11	84.44	in	2,572	21	56	76.67
15	10	but	741	55–62	11	84.44	.	1,776	21	51	73.33
16	9	that	533	55–62	11	84.44	of	1,070	21	41	73.33
17	8	if	377	55–62	11	84.44	for	578	12	63	66.67
18	7	?	255	55–62	11	84.44	to	282	5	63	60.00
19	6	and	171	55–62	11	84.44	from	141	5	42	57.78
20	5	by	93	55–62	10	84.44	what	48		48	41.11
21	4	:	41	1–20	21	78.89	with	22	19	2	35.56
22	3	as	24	1–31	9	65.56	at	14	19	2	35.56
23	2	on	12	1–55	4	61.11	-	3	12	2	35.56
24	1	;	10	1–55	4	61.11	(				
25	0	not					,				

Columns present parameters: (a) elimination stage, (b) number of characteristic features left, (c) attribute eliminated at this stage, (d) number of all rules in a decision algorithm, (e) minimal support required of rules resulting in maximal classification accuracy, (f) minimal number of rules meeting constraints, and (g) maximal classification accuracy (%)

Application of ANN Ranking in reduction of FDA results in rather steep decrease in the number of remaining decision rules, while the classifiers predict with the same or only slightly reduced accuracy. Reversed ANN Ranking brings much slower algorithm reduction, but the performance is worsened instantly and irreparably.

**Fig. 6:**
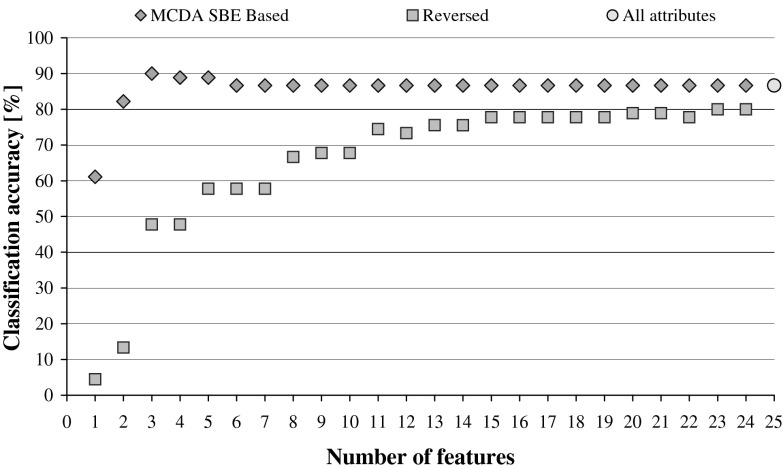
Reduction of all rules on examples decision algorithm while following DRSA feature ranking and its reverse

As establishing of DRSA Ranking through sequential backward elimination with generation of minimal cover decision algorithms is treated as a separate process, this ranking can also be used in the procedure of decision rule ranking and reduction, limiting all rules on examples algorithm, the results of which are given in Table 5 and the performance shown in Fig. 6.

**Table 5:** Backward elimination of decision rules from all rules on examples (FDA) algorithm induced for all features, with following DRSA Ranking of attributes and its reverse

		DRSA Ranking (SBE for MCDA)					Reversed				
(a)	(b)	(c)	(d)	(e)	(f)	(g)	(c)	(d)	(e)	(f)	(g)
1	24	and	47,064	66	17	86.67	not	61,382	48	49	80.00
2	23	!	37,662	66	16	86.67	by	47,968	48	43	80.00
3	22	,	32,655	62	20	86.67	as	37,258	45	43	77.78
4	21	?	27,671	62	20	86.67	in	27,552	44	54	78.89
5	20	what	21,473	62	20	86.67	for	20,377	44	46	78.89
6	19	:	19,736	62	20	86.67	;	18,047	33	27	77.78
7	18	.	14,716	62	20	86.67	from	13,423	33	24	77.78
8	17	of	10,964	62	20	86.67	on	11,763	33	24	77.78
9	16	that	8,575	62	20	86.67	with	8,661	33	24	77.78
10	15	(	6,751	62	20	86.67	-	7,603	33	24	77.78
11	14	this	4,907	59	23	86.67	to	5,324	24	49	75.55
12	13	but	3,440	59	23	86.67	at	3,924	24	49	75.56
13	12	if	2,462	59	23	86.67	if	2,880	25	41	73.33
14	11	at	1,795	59	23	86.67	but	1,950	18	75	74.44
15	10	to	1,208	59	23	86.67	this	1,195	11	113	67.78
16	9	-	854	59	23	86.67	(	858	11	109	67.78
17	8	with	624	59	23	86.67	that	594	11	84	66.67
18	7	on	533	59	23	86.67	of	312	13	44	57.78
19	6	from	335	59	20	86.67	.	205	9	40	57.78
20	5	;	209	13	79	88.89	:	162	9	40	57.78
21	4	for	107	10	63	88.89	what	85	8	25	47.78
22	3	in	65	10	46	90.00	?	58	8	25	47.78
23	2	as	35	10	30	82.22	,	26	3	16	13.33
24	1	by	10	55	4	61.11	!	4	4	1	4.44
25		not					and				

Columns list parameters: (a) elimination stage, (b) number of characteristic features left, (c) attribute eliminated at this stage, (d) number of remaining rules without constraints, (e) minimal support required of rules to arrive at the highest classification accuracy, (f) number of rules meeting constraints on support, and (g) maximal classification accuracy (%)

The tendencies visible in predictive accuracy for reduced decision algorithms while following DRSA Ranking and its reverse directly remind these observed previously in the wrapper mode when the ranking was established. The procedures enable to filter out these rules from FDA algorithm which contain conditions on irrelevant attributes and return algorithms with significantly decreased number of decision rules while maintaining or even increasing the classification accuracy.

**Fig. 7:**
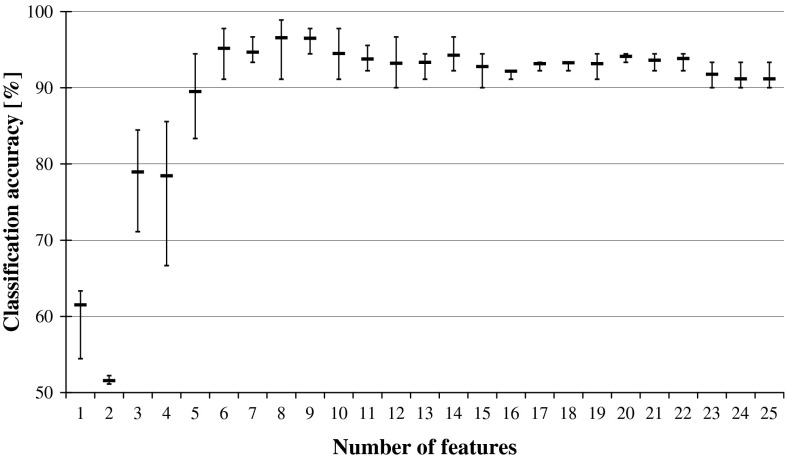
Reduction of characteristic features for ANN classifier while following DRSA Ranking. The predictive accuracy is plotted in relation to the number of features, and for each average, there is indicated maximal and minimal performance

**Fig. 8:**
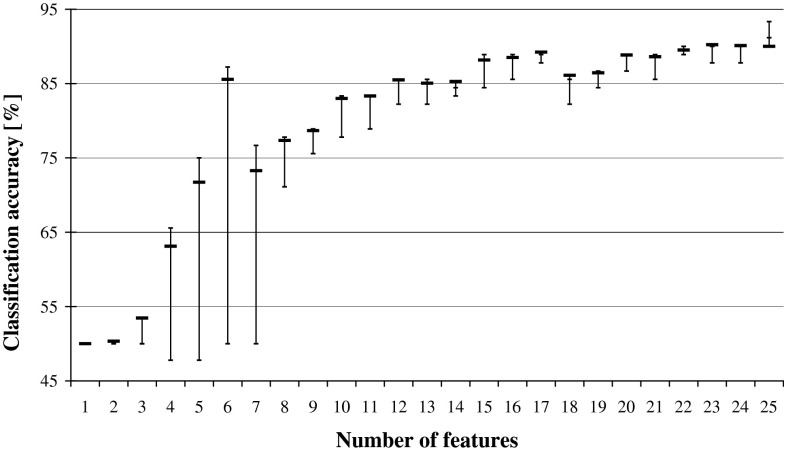
Reduction of characteristic features for ANN classifier while following the reversed DRSA Ranking. The predictive accuracy is plotted in relation to the number of features, and for each average, there is indicated maximal and minimal performance

When DRSA Ranking was employed in reduction of input characteristic features to the artificial neural network, it resulted in yet another hybrid solution. At each elimination stage, a single feature was disregarded and the influence of it on the network performance studied, as plotted in Fig. 7. When the reversed ranking is exploited (Fig. 8), comparison of these two graphs reveals very close resemblance to the one displayed in Fig. 1, illustrating the performance of DRSA wrapper employing SBE.

From all tested combinations of wrappers, the best performance was displayed for ANN classifiers employing DRSA Ranking in backward elimination of features (Fig. 7). Good results were also obtained in reduction of all rules on examples algorithm generated for all features, while following DRSA Ranking (Table 5; Fig. 6). In this case, however, this can be explained by the wrapper bias when two systems of the same type, sharing the same characteristics, are combined. The same cannot be stated for the former case, as the differences between DRSA and ANN classifiers are clearly shown in the observed process of sequential backward elimination of features, resulting in two distinctively different rankings.

Using ANN Ranking in backward attribute reduction and then inducing new rules and algorithms for all rules on examples enables to discard eight variables (32 %) before the performance starts decreasing (Table 3; Fig. 4). ANN Ranking in FDA reduction brings also rejection of eight variables and as many as 51,888 decision rules (83 %). Application of reversed rankings, both DRSA- and ANN-based, always resulted in worsened performance.

### Results for waveform dataset

**Fig. 9:**
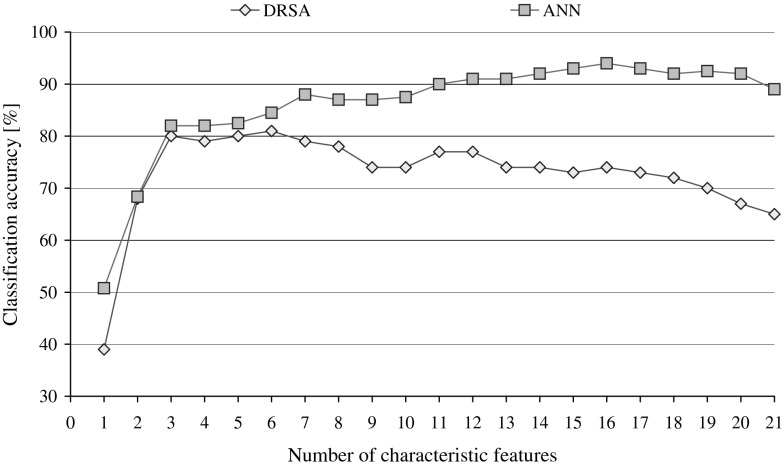
Performance of DRSA and ANN classifiers observed in the sequential backward elimination of characteristic features

The attributes for the waveform dataset are not described in detail in the UCI ML repository; therefore, for convenience, they were simply labelled form a1 to a21 and the two decision classes corresponded to the selected wave types, type 0 and type 1. The two rankings obtained by sequential backward elimination for DRSA and ANN classifiers are given in Table 6, with details of induced algorithms and performance for both systems, which is also plotted for both types of classification systems in Fig. 9.

**Table 6:** Backward elimination of attributes basing on the performance of DRSA and ANN classifiers for waveform dataset

						DRSA Ranking		ANN Ranking	
(a)	(b)	(c)	(d)	(e)	(f)	(g)	(h)	(g)	(h)
0	21	55		3	20	65	a2	89	a2
1	20	56				67	a18	92	a19
2	19	50				70	a20	92	a10
3	18	49		2	30	72	a6	93	a12
4	17	50		2	35	73	a16	94	a17
5	16	52		2	31	74	a15	93	a13
6	15	54		4	17	73	a19	92	a8
7	14	54		2	33	74	a12	91	a5
8	13	52		3	32	74	a13	91	a21
9	12	49		2	20	77	a8	90	a7
10	11	53		2	35	77	a3	87	a14
11	10	48		2	35	74	a21	87	a4
12	9	48		3	24	74	a17	87	a1
13	8	45		3	27	78	a14	88	a3
14	7	31	28	5	17	79	a9	84	a20
15	6	30	21	8	10	81	a5	82	a18
16	5	36	24	6	13	80	a1	82	a16
17	4	33	20	9	11	79	a4	82	a6
18	3	34	13	4	12	80	a7	68	a15
19	2	40	6			68	a11	51	a11
21	1	3	2			39	a10	50	a9

Columns present parameters: (a) elimination stage, (b) number of characteristic features left, (c) number of rules in DRSA minimal cover decision algorithm without any constraints, (d) number of exact rules when they are fewer than the total number, (e) minimal support required of DRSA rules resulting in maximal classification accuracy, (f) number of exact DRSA rules meeting constraints on support, (g) predictive accuracy of the classifier (%), and (h) attribute selected to be eliminated

The performance of classifiers is compared against each other and to the reference point constituted by the predictive accuracies obtained for the complete set of 21 attributes. Minimal cover decision algorithm induced classified only 65 % with 55 rules limited to 20 by constrains on support to be equal at least 3. All rules on examples algorithm achieves 74 % recognition ratio (31,718 rules constrained to 58 for support equal or higher than 48). ANN with 21 input features recognised correctly 89 % of testing samples.

**Fig. 10:**
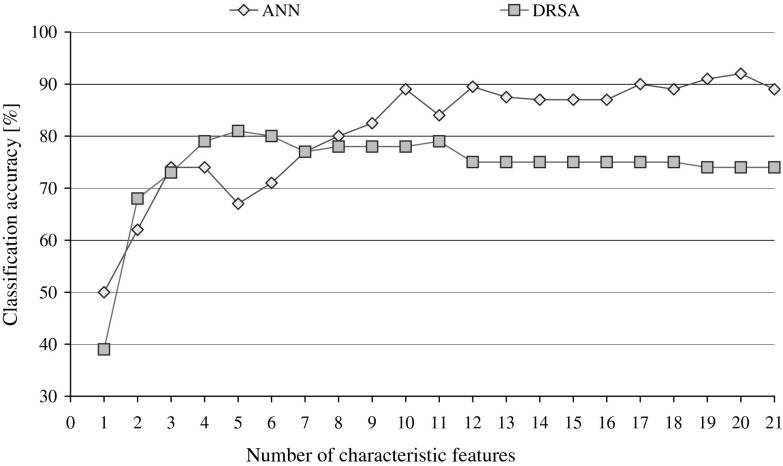
Pruning of inputs for ANN classifier compared to pruning of rules from all rules on examples decision algorithm induced for the complete set of attributes, with dimensionality reduction executed while following DRSA Ranking

When DRSA Ranking of features is applied for systematic reduction of inputs to connectionist classifiers, in the initial phase some increase in performance can be observed (see Fig. 10), yet the visible trend is not strictly monotonic. The same ranking is also employed for reduction of selected rules from all rules on examples algorithm in the procedures described before and in this process significant gains can be observed: we can reduce 17 out of 21 attributes (close to 81 %) and still have increased performance. This, however, comes without surprise as both inducers share the same general characteristics, hence the resulting bias.

Imposing ANN Ranking on DRSA processing is performed again in two ways: either for the gradually decreasing subsets of conditional attributes new decision rules are induced (both MCDA and FDA), or the set of rules from the previously inferred complete decision algorithm is analysed and some rules rejected when they refer to discarded features. The details of all resulting solutions are given in Table 7. From the observed performance, we can detect that for all rules on examples, it is possible to reject 13 out of 21 conditional variables (almost 62 %), while not only the recognition is not worse, but increased.

**Table 7:** Backward elimination of conditional attributes using ANN Ranking with induction of new decision algorithms and with reduction of full decision algorithm previously inferred

		Induction of DA after attribute elimination										Reduction of rules from FDA			
		Minimal cover DA					All rules on examples DA								
(a)	(b)	(c)	(d)	(e)	(f)	(g)	(c)	(d)	(e)	(f)	(g)	(c)	(e)	(f)	(g)
1	20	56				67	25,176		48	58	75	25,176	48	58	74
2	19	56		2	33	64	20,041		48	58	74	20,041	48	58	74
3	18	61		2	36	64	15,909		37	97	71	15,909	37	97	73
4	17	59		2	39	58	12,177		33	95	76	12,177	33	95	76
5	16	54		2	30	61	9,872		33	95	76	9,872	33	95	76
6	15	53				64	6,835		33	90	76	6,835	33	90	76
7	14	55		2	32	63	4,925		18	211	75	4,925	18	211	75
8	13	53		3	18	64	3,408		18	185	75	3,489	18	186	75
9	12	58				67	2,235		10	269	75	2,298	10	272	75
10	11	64		2	40	68	1,388		11	212	78	1,444	11	215	78
11	10	60				68	976		11	184	78	1,028	11	186	78
12	9	62	60	3	26	66	796	635	11	137	78	672	11	139	78
13	8	56	46	3	23	64	1,090	340	11	97	75	368	11	101	75
14	7	51	37			66	942	187	25	45	71	230	26	46	71
15	6	51	37			67	473	135	5	78	73	166	5	92	73
16	5	46	33			68	271	101	5	65	73	130	5	81	73
17	4	49	31			68	145	68	5	50	69	90	5	65	70
18	3	37	16			70	47	24	14	21	67	45	17	29	68
19	2	32	11	8	10	68	33	18	17	18	67	33	17	27	67
20	1	3	2			30	3	2			30	7	26	4	30

Columns present parameters: (a) elimination stage, (b) number of characteristic features left, (c) number of all rules in a decision algorithm, (d) number of exact rules in a decision algorithm when they are fewer than the total number of rules, (e) value for minimal support required of rules resulting in maximal classification accuracy, (f) minimal number of rules meeting constraints, and (g) maximal classification accuracy (%)

When all rules on examples decision algorithms (a new one and the reduced FDA) are compared in each stage, it becomes apparent that they are in fact very close. Even though the numbers of rules involved are not always exactly the same, the resulting classification accuracy is almost identical, which suggests choosing the second way, that is with reduction of FDA generated for the complete set of features instead of inducing new algorithms. It requires significantly less effort as the hard part of computations is already executed. Once some kind of method for pruning of rules is established, its execution could be less demanding than the induction process.

For comparison, also some tests for reversed rankings were performed, with discarding the least ranking attributes, but results were worse when compared to the corresponding solution for most ranking variables, with differences depending on the number of elements reduced, often increasing along with it.

All experiments conducted, for both stylometric and waveform datasets, confirm the usefulness of the proposed methodology of combining wrappers for estimation of feature relevance used next it their backward reduction.

## Conclusions

Filter and wrapper are two approaches to selection and reduction of characteristic features, which can be used as a way to observe their relevance or redundancy for the considered classification task. Filters work independently on the particular learning system employed for pattern recognition, while wrappers condition the choice of attributes on performance of the classifier. When a wrapper is used to establish a ranking of characteristic features in a separate process, it can be treated as a filter for another classification system. The paper presents a methodology that involves a combination of wrapper approaches, applied to observe relevance of characteristic features for two binary classification tasks with balanced data.

In the pre-processing stage of the wrapper mode, minimal cover decision algorithms inferred in DRSA and artificial neural networks with MLP topology are used to establish two rankings of the studied features through their sequential backward elimination. The resulting orderings are next employed as filters for inputs to new inducers, of the same and different type. Only application of reversed rankings resulted in worsened performance, while for all other cases, there were several alternative smaller subsets of variables for which the classification accuracy was at the same or increased level.

As the primary classification task authorship attribution was executed, which belongs with computational stylistics—a study of writing styles that requires observations of linguistic habits and preferences and employs stylometric characteristic features. For verification, the same reduction procedures were applied to another dataset, taken from UCI Machine Learning Repository. The results from the conducted experiments for both datasets show similar trends in performance in perspective of dimensionality reduction which validates the proposed research framework.
